# Hypervalent Nonbonded Interactions of a Divalent Sulfur Atom. Implications in Protein Architecture and the Functions

**DOI:** 10.3390/molecules17067266

**Published:** 2012-06-13

**Authors:** Michio Iwaoka, Noriyoshi Isozumi

**Affiliations:** Department of Chemistry, School of Science, Tokai University, Kitakaname, Hiratsuka-shi, Kanagawa 259-1292, Japan

**Keywords:** Protein Data Bank, chalcogen bonds, σ-hole bonds, molecular assembly, protein engineering, drug design

## Abstract

In organic molecules a divalent sulfur atom sometimes adopts weak coordination to a proximate heteroatom (X). Such hypervalent nonbonded S···X interactions can control the molecular structure and chemical reactivity of organic molecules, as well as their assembly and packing in the solid state. In the last decade, similar hypervalent interactions have been demonstrated by statistical database analysis to be present in protein structures. In this review, weak interactions between a divalent sulfur atom and an oxygen or nitrogen atom in proteins are highlighted with several examples. S···O interactions in proteins showed obviously different structural features from those in organic molecules (*i.e.*, π_O_ → σ_S_* *versus* n_O_ → σ_S_* directionality). The difference was ascribed to the HOMO of the amide group, which expands in the vertical direction (π_O_) rather than in the plane (n_O_). S···X interactions in four model proteins, phospholipase A_2_ (PLA_2_), ribonuclease A (RNase A), insulin, and lysozyme, have also been analyzed. The results suggested that S···X interactions would be important factors that control not only the three-dimensional structure of proteins but also their functions to some extent. Thus, S···X interactions will be useful tools for protein engineering and the ligand design.

## 1. Introduction

**Figure 1:**
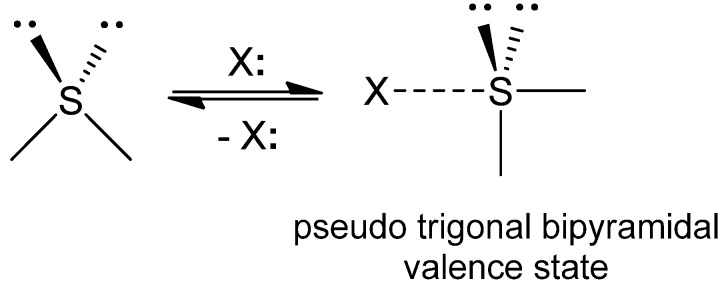
Hypervalent nonbonded S···X interactions of a divalent sulfur atom.

A sulfur atom is usually present in a divalent state in organic molecules. However, it sometimes adopts a weak coordination to a proximate heteroatom (X), such as O, N, and S, in solution as well as in the solid state [1,2,3,4]. Such hypervalent weak atomic interactions, so-called nonbonded S···X interactions, are of significant interest because they can control not only the molecular assembly of organic sulfur compounds in advanced materials with unique conductivity or optical properties [5,6] but also the structure and reactivity of isolated molecules [7,8,9]. The hypervalent (or out-of-octet) nature of a divalent sulfur atom is physic-chemically explained on the basis of the observed apparent directionality by coordination of the lone pair of a ligand (X) to the sulfur center to obtain the pseudo trigonal bipyramidal valence state (Figure 1). Apart from the proposed orbital interaction [10], importance of other factors, such as electrostatic interaction and electron correlation, has also been pointed out for formation of the weak nonbonded interaction [7,11]. Recently, variation of S···X interactions is extending to two-center-three-electron S.˙.N^+^ [12,13], bifurcated SS···O [14], and S···CH_2_(carbene) [15] interactions.

**Figure 2:**
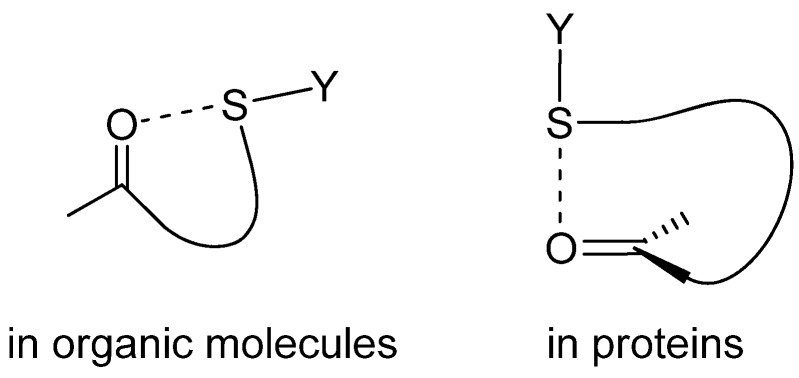
Nonbonded S···X interactions in organic molecules and proteins.

On the other hand, the sulfur atoms of cystine (SSC-type) and methionine (CSC-type) residues in proteins are usually considered as hydrophobic groups, forming no specific nonbonded interaction. However, the recent statistical analyses using the structure database have clearly demonstrated that similar hypervalent S···X interactions to those characterized in organic molecules are widely present in protein structures [16,17,18]. Interestingly, the directionality of the S···O interactions found in proteins is obviously different from that observed in organic sulfur compounds (*i.e.*, n_O_ → σ_S_* *versus* π_O_ → σ_S_* as shown in Figure 2) [19], although in both cases the S atom adopts electron coordination in the backside of the S–Y bond (*i.e.*, in the σ hole). Importance of this non-classical atomic interaction of proteins in the stability, function, and evolution has also been pursued by us [18,19,20] and other groups [16,21,22,23]. 

In this review, structural features and functional aspects of weak nonbonded S···X interactions in proteins are highlighted with several examples. In the next two sections, S···X interactions in organic molecules and in proteins are overviewed, respectively. Structural features of the S···X interactions in proteins are discussed in comparison with those observed in organic molecules. In the subsequent sections, the S···X interactions in proteins are implicated in protein architecture and the functions based on the results from structure database analyses using a large set of heterogeneous protein structures and rather small sets of structures of four model proteins, *i.e.*, phospholipase A_2_ (PLA_2_), ribonuclease A (RNase A), insulin, and lysozyme. It is strongly suggested that the S···X interactions are important factors that control protein structures and functions to some extent. Perspectives of the research on such weak nonbonded interactions are given in the Conclusions. Throughout this review, the term S···X interactions is used for weak hypervalent atomic interactions, although other terminologies, such as σ-hole bonds [24] and chalcogen bonds [25], are frequently used in the recent literature.

## 2. S···X Interactions in Organic Compounds

### 2.1. Database Analysis

Attractive interactions between a divalent sulfur atom (S) and nearby heteroatoms (X) have been well recognized in small organic compounds. In 1977, Rosenfield *et al.* [2] surveyed close S···X contacts in organic and inorganic crystals using the Cambridge Crystallographic Database [26] and found an obvious directional preference of X with respect to S, as shown in Figure 1. The directionality was reasonably explained by the presence of specific nonbonded S···X interactions. On the other hand, the directional preference of the S···O interactions with respect to O was studied in detail by Kucsman and Kapovitz [27]. For intramolecular 1,4- and 1,5-type S···O=C interactions, the S atom tended to lie in the direction of the O lone pairs (*i.e.*, the n_O_ direction) rather than the π electrons (*i.e.*, the π_O_ direction). These statistical analyses using the structure database demonstrated the importance of the n_O_ → σ_S_* orbital interaction for formation of S···O interactions in organic molecules.

A similar database analysis for analogous nonbonded S···S interactions was carried out by Row and Parthasarathy in 1981 [3]. They reported that S···S interactions in organic crystals would be stabilized by the orbital interaction between the lone pair of one sulfur atom (n_S_) and the anti-bonding orbital of the other sulfur atom (σ*_S_) as observed in the S···O interactions. Desiraju and Nalini [4] obtained a similar donor-acceptor interaction mechanism of S···S interactions. The presence of S···C(π) interactions in organic crystals was also suggested by Zauhar *et al.* [28].

### 2.2. Energetic Elements of S···X interactions

Database analyses for various types of nonbonded S···X (X = O, S,* etc.*) interactions clearly demonstrated that specific directional preferences are present for the S···X interactions in organic crystals. The directionality strongly indicated the importance of the orbital interaction between the interacting atoms for the stability. For instance, S···O and S···S interactions would be stabilized by the n_O_ → σ_S_* and n_S_ → σ_S_* orbital interactions, respectively. However, the nonbonded distances between the two interacting atoms are sometimes only marginally shorter than the sum of the van der Waals radii [29], and, in such cases, the directionality becomes rather subtle. Therefore, there should be other electronic factors that are contributing to formation of the S···X interactions.

The electrostatic nature of the 1,4-type S···O interactions between a positively charged S atom and a negatively charged ethereal O atom was suggested by Burling and Goldstein [7] based on the substituent effects and the theoretical calculations. Dahaoui *et al.* [11] pointed out importance of van der Waals forces (or electron correlation effects) for the S···S interactions. Thus, several energetic elements, such as orbital interaction, electrostatic interaction, and electron correlation, must be considered for fully understanding S···X interactions. Recent sophisticated theoretical analyses applying the atoms-in-molecules (AIM) method by Nakanishi [30] and the symmetry-adapted perturbation theory (SAPT) by Scheiner [31,32] provided more exact description of S···X interactions in terms of the total electron energy density and Laplacian of the electron density at the bond critical points of AIM as well as the electrostatic, induction, and dispersion components of SAPT.

### 2.3. Examples

**Figure 3:**
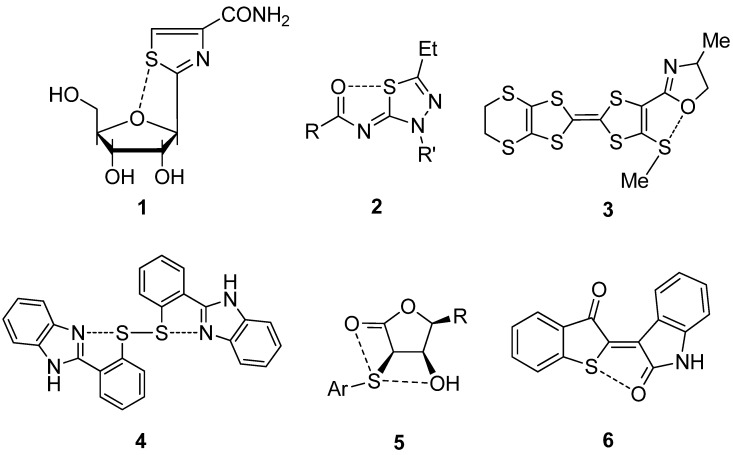
Examples of S···O interactions in organic molecules.

Intramolecular nonbonded S···X interactions have been extensively studied for some organic sulfur compounds in relation to the biological activities as well as the physical properties as advanced materials. Examples are shown in Figure 3. 

Burling and Goldstein [7] demonstrated the importance of an intramolecular 1,4-type S···O interaction of thiazole nucleoside analogues (**1**) for their antitumor activity. Nagao [8,33] reported that 1,5-type S···O interaction plays important roles in the antagonism of (acylimino)thiadiazoline derivatives (**2**) towards an angiotensin II receptor. Similar S···O and S···N interactions are responsible for the molecular structures and functions of TTF-oxazoline derivatives (**3**) [6], bis[2-(1*H*-benzimidazol-2yl)phenyl]disulfide (**4**) [34], β-hydroxy-α-sulfenyl-γ-butyrolactones (**5**) [35], and thioindirubin (**6**) [36].

## 3. S···X Interactions in Proteins

Weak nonbonded interactions are important physicochemical forces that control the structure of proteins [37]. Ionic interaction, hydrogen bond, van der Waals forces, and hydrophobic interaction are mainly considered as this class of interactions, but some novel interaction patterns, such as C–H···O hydrogen bond [38,39,40], cation-π interaction [41,42,43], and CH/π hydrogen bond [44,45,46], have recently been characterized in folded protein structures. Importance of these new interactions for the stability and functions of proteins has also been pointed out. The S···X interactions can be another member of such non-classical interactions.

The SSC and CSC groups involved in cystine and methionine residues, however, were usually considered just as hydrophobic moieties in folded protein structures until recently, except for S···C(π) interactions [47] and weak NH···S and OH···S hydrogen bonds [48]. The S···C(π) interactions in proteins were first pointed out by Morgan and co-workers [49], who analyzed the close atomic contact between S and a π-plane in eight protein structures and found that the S atoms have a propensity to come over the π-plane. On the other hand, Reid *et al.* [50] suggested by using a larger set of protein structures that the close S···C(π) contact in proteins can also be explained by CH···S interactions because the S atoms access to the π-plane from the side rather than the top. According to several experimental and theoretical studies having been reported to date [22,28,51,52], however, the nature of S···C(π) interactions in proteins would be well rationalized by the interaction between the aromatic π electrons and the S atom [22,47]. Meanwhile, NH···S and OH···S hydrogen bonds were suggested to play some roles in particular proteins [48], but the interactions were rarely found in protein structures. The S atoms of cystine and methionine would have only a weak character of a hydrogen-bond acceptor.

### 3.1. Database Analysis

Nonbonded S···X interactions in proteins have recently been pursued by several research groups [16,17,18,21,22,23,18,21]. The stereochemistry of the nonbonded S(CSC)···O interactions for methionine residues was first analyzed by Carugo [21] using a small set of protein structures. Although no strong directional preference was observed, the result suggested that the S···O interactions in proteins would have either a very weak or physicochemically different character from those observed in small molecules. On the other hand, Iwaoka* et al.* [17] thoroughly surveyed close S···X (X = O, N, S, C,* etc.*) atomic contacts involved in 604 high-resolution (≤2.0 Å) heterogeneous X-ray structures selected from the Protein Data Bank [53]. Statistical analyses of the relative nonbonded S···X distance (*d* = *r*_S···X_ − *vdw*_S_ − *vdw*_X_), the directionality around the S and X atoms, and the location along the amino acid sequence revealed distinct structural features of the S···X interactions. In case of the most frequent S···O interactions, both SSC and CSC S atoms tend to approach a main-chain O atom perpendicularly to the amide plane (the π_O_ direction), and the O atom tends to approach the S atom from the backside of the S–S or S–C covalent bonds (the σ_S_* direction). Similar directionalities of the S(CSC)···O interactions were also reported by Pal and Chakrabarti [16]. The structural propensity observed is in striking contrast to the S···O interactions in organic crystals, in which the n_O_ orbital, not π_O_, is usually used to form the interaction.

Table 1 shows numbers of S(SSC)···X contacts in selected protein structures [17]. For short contacts (*d* ≤ 0.0 Ǻ), a probability of S···O contacts increases significantly, suggesting the presence of specific S···O interactions in proteins. According to the statistical analysis for the obtained data, four types of nonbonded S···X interactions have been clearly characterized, *i.e.*, S–S···O=C, C–S···O=C, C–S···N, and S–S···S–S interactions [18].

**Table 1:** Numbers of S(SSC)···X contacts in selected proteins [17] *^a^*.

X =	*d ^b^* ≤ 0.0 Ǻ	0.0 < *d* ≤ 0.5 Ǻ
O	100	664
N	33	359
S	15	68
C	134	1478
Others	2	0
Total	284	2569

*^a^* 604 heterogeneous proteins with high resolution (≤2 Ǻ) were selected. The total number of Cys residues was 790; *^b^* A relative nonbonded S···X distance defined as *d* = *r*_S···X_ − *vdw*_S_ − *vdw*_X_.

A majority of close S···O contacts for an SSC group in proteins is assigned to S–S···O=C interactions, which can be characterized by the linear S–S···O atomic alignment and the vertical access of the S atom to the carbonyl plane as shown in Figure 2. According to the directionality, a significant contribution from the π_O_ → σ_S_* orbital interaction is obvious. The S–S···O=C interactions are most frequently observed in helices, suggesting that the S···O interactions would support the stability. The C–S···O=C interaction formed between a methionine side-chain and a main-chain peptide group has a similar character to the S–S···O=C interaction, but the strength of the interaction is weaker with attenuated directionality. Similarly, the C–S···N interaction between a methionine side-chain and a main-chain peptide group can also be characterized by the linear C–S···N atomic alignment and the vertical access of the S atom to the amide plane, suggesting a contribution from the π_N_ → σ_S_* orbital interaction. On the other hand, most of close S···S contacts in proteins can be assigned to S–S···S–S interaction, which would be stabilized by the n_S_ → σ_S_* orbital interaction in a similar manner to that observed in organic crystals [3].

### 3.2. Energetic Elements of S···X Interactions

**Figure 4:**
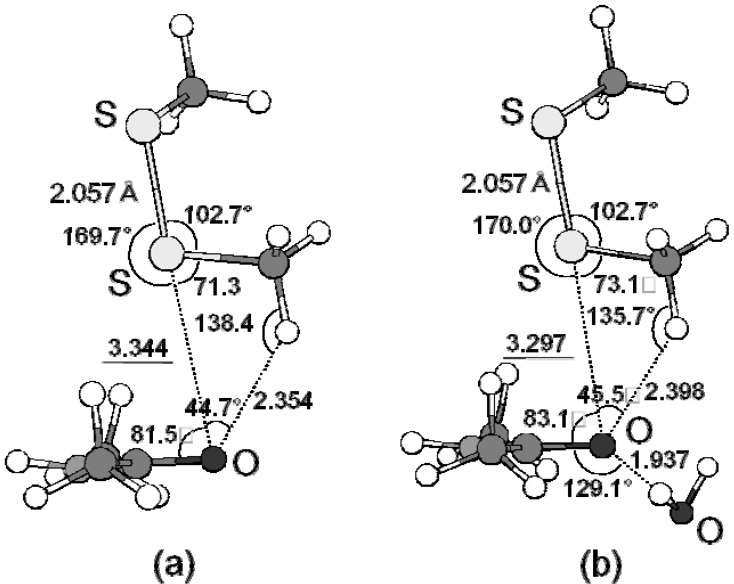
Optimized structures of the complexes between CH_3_SSCH_3_ and CH_3_CONHCH_3_ without H_2_O (**a**) or with H_2_O (**b**) at MP2/6-31G(d) [17].

*Ab initio* calculation was carried out for the model complexes (CH_3_SSCH_3_ + CH_3_CONHCH_3_ and CH_3_SCH_3_ + CH_3_CONHCH_3_) to investigate the nature of the S···X interactions observed in proteins [17,18]. The calculation using the Møller-Plesset method (MP2) [54] suggested the importance of the dispersion force or the electron correlation for the stability of the S···X interactions. The S–S···O=C interaction was estimated to be as strong as 3.2 kcal/mol at MP2/6-31G(d) and would be predominantly stabilized by electron correlation with a significant contribution from the π_O_ → σ_S_* orbital interaction (Figure 4). The presence of a CH···O hydrogen bond and an additional hydrogen bond (such as NH···O or OH···O) at the main-chain carbonyl O atom would stabilize the complex cooperatively with the S···O interaction. On the other hand, the C–S···O=C and C–S···N interactions should be weaker than the S–S···O=C interaction with strengths up to 2.5 and 2.9 kcal/mol, respectively.

**Figure 5:**
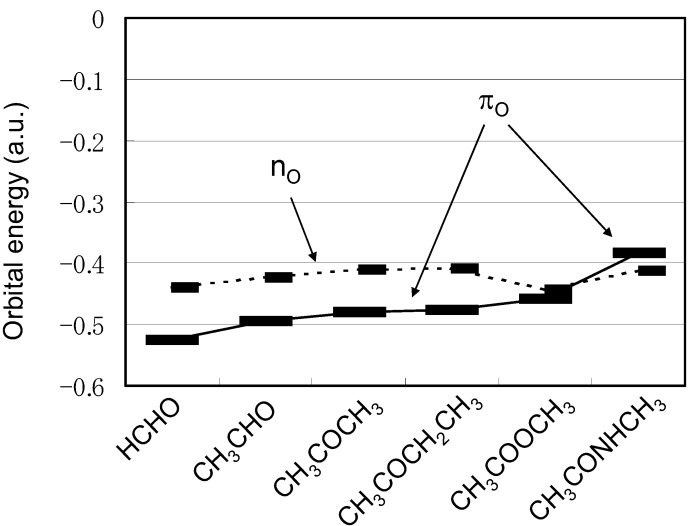
Energy levels of n_O_ and π_O_ orbitals for various carbonyl compounds calculated at MP2/6-31G(d) [19].

The reason for the observed discrepancy in the directionality between the S···O interactions in proteins and organic molecules can be explained on the basis of the HOMO levels of various carbonyl compounds [19]. As graphically shown in Figure 5, for most of the carbonyl compounds the HOMO is assigned to the oxygen lone pair (n_O_) lying in the carbonyl plane rather than the π orbital expanding perpendicular to the plane. 

However, the π_O_ level of amide CH_3_CONHCH_3_ is remarkably raised compared with other carbonyl compounds, while the n_O_ level remains almost unchanged. The elevation of the π_O_ orbital would be due to the conjugation between the N lone pair and the carbonyl group. Thus, inversion of the energy levels of n_O_ and π_O_ would be responsible for the observed directional preferences of the S···O interactions in proteins.

### 3.3. Examples

The importance of S···O interactions in the enzymatic function of proteins has been pointed out for some particular cases. Taylor and Markham [55] suggested that the electrostatic S···O interaction between the S atom of a methionine substrate and the carboxylate O atom of Asp118 plays a major role in the enzymatic activity of S-adenosylmethionine synthetase. Brandt* et al.* [56] reported that the cleavage of a disulfide bond in the extracellular region of the G-protein receptors, which is an important process for the receptor activation, is catalyzed by the S···O interaction between Cys121 and the carboxylic group of Asp288. The existence of S···O interactions was suggested for the complexes between *N*-acetylglucosamine-thiazoline and β-hexosaminidase and between benzophenone and porcine odorant-binding protein [33]. Importance of the possible S···O interaction between the Met10 and Thr317 residues of adenylosuccinate lyase for the stability of the protein structure was also suggested using the M10L mutant [57]. More recently, the S···N interactions between an OSCN^−^ ligand and the imidazole ring of His109 in lactoperoxidase [58] and between the sulfenic acid form of Cys50 and the imidazole ring of His42 in peroxiredoxin [59] were suggested to play roles in the functions.

## 4. Implications of S···X Interactions in Protein Architecture

**Figure 6:**
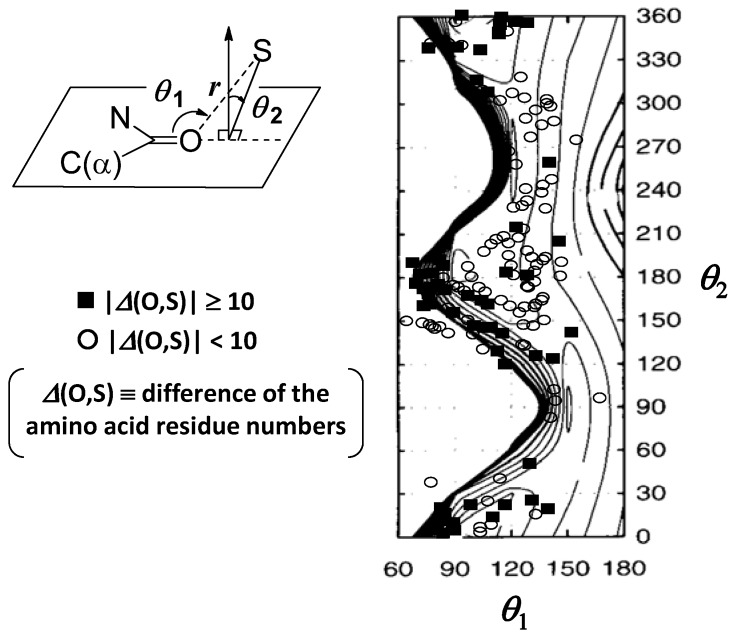
The directionality around the O atom in the S–S···O=C interactions in proteins superimposed on the potential surface calculated for the complex of between CH_3_SSCH_3_ and CH_3_CONHCH_3_ at MP2/6-31G(d). The contour lines are drawn with an interval of 0.25 kcal/mol. This figure was modified from reference [18].

According to the comparison between the results from database analyses and ab initio calculation, it was clear that the directional preferences of the S···O interactions observed in protein structures are in accord with the profiles of the potential surfaces calculated for the isolated model complexes [18]. An example is shown in Figure 6 for the case of the directionality around the O atom in the S–S···O=C interactions in proteins. The remarkable agreement strongly suggested that these nonbonded interactions are important determinants for protein architecture. Similar phenomena have been found between the Ramachandran plots of the amino acid residues and the single amino acid potentials (SAAP) in water, which has already been applied to developing a new all-atom force field for molecular simulation of peptide molecules [60,61].

Protein structures are generally considered to be flexible because they are governed only by weak nonbonded interactions. Therefore, coincidence of the statistical conformational preference for the interaction of proteins with the potential surface calculated for the isolated model is to be noticed and would have important implications in protein architecture. The detailed analysis revealed the following features [19]. The linearity of Y–S···O (Y = S or C) alignment in both organic molecules and proteins is not affected by the crystal packing force but can be disturbed by the structural constraint between the interacting fragments. On the other hand, the vertical nature of Y–S···O=C interactions is not affected by the presence of other weak nonbonded interactions in protein structures

**Figure 7:**
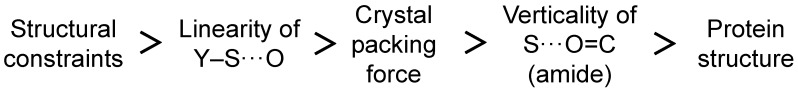
The factors that control molecular structure of organic molecules and proteins in the solid state.

The directionality, however, is easily affected by crystal packing force for organic molecules. Thus, the order of the factors that control molecular structure of organic molecules and proteins in the solid state can be summarized as shown in Figure 7 [19]. 

The O atom has strong tendency to approach the S atom from the backside of the S–C or S–S bond (in the σ_S_* direction), irrespective of the types of carbonyl groups. On the other hand, the S atom tends to approach the O atom either within the carbonyl plane (in the n_O_ direction) or from the vertical direction (in the π_O_ direction). In the case of S···O(amide) interactions, the vertical direction is significantly preferred, due probably to elevation of the π_O_ orbital. The linearity of the S···O interactions in organic molecules would overcome the crystal packing force, whereas the vertical nature of the S···O(amide) interactions may be affected by the packing force. The verticality, however, would survive in protein structures. These structural features will be informative for protein engineering and molecular design of functional organic sulfur compounds.

## 5. Implications of S···X Interactions in Protein Functions

With a success in characterization of S···X interactions in proteins, we subsequently sought out particular protein families or domains, for which specific S···X interactions are commonly present in a wide range of the structures registered in the Protein Data Bank [53]. Phospholipase A_2_ (PLA_2_) was the first example of such proteins [20]. We have thus far found three more examples by applying the same method of the database analysis that was applied for the PLA_2_ family. The results are summarized in this section.

### 5.1. Phospholipase A_2_ (PLA_2_) [20]

**Figure 8:**
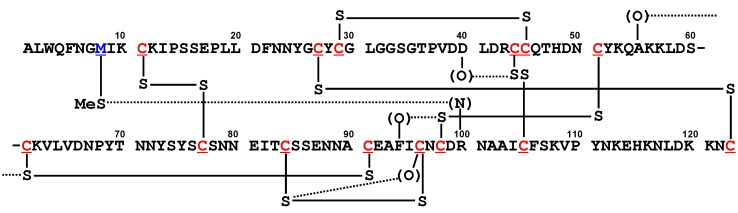
The amino acid sequence of bovine PLA_2_ with notations of common S···X interactions.

PLA_2_ [62,63], a small globular protein consisting of about 130 amino acid residues, is a SS-rich enzyme that catalyzes hydrolysis of the 2-acyl ester bond of phosphoglycerides in the presence of a calcium ion. There are two domain groups in the vertebrate PLA_2_ family; PLA_2_ and snake PLA_2_ (sPLA_2_). Comprehensive search for close S···X (X=O, N, and S) contacts in the structures of the PLA_2_ domain, which were retrieved from protein data bank, revealed the presence of four common S···O interactions, *i.e.*, S(C44)···O(D40), S(C61)···O(A55), S(C84)···O(C96), and S(C98)···O(F94), and one common S···N interaction, *i.e.*, S(M8)···N(R100), as shown in Figure 8. Most of the S···X interactions were found in the vicinity of the active site and to tolerate the conformational changes caused by binding of the substrate. It was reported in the literature that the enzymatic activity of porcine PLA_2_ is decreased in the M8,20L mutant [64]. This would be explained by elimination of the S(M8)···N(R100) interaction.

On the other hand, an evolutional aspect of the S···X interactions was analyzed for the sPLA_2_ domain. For this domain group, the phylogenetic dendrogram was already analyzed by Ohno *et al.* [65] using the amino acid sequences of various PLA_2_ involved in venom of snakes inhabiting the southern islands of Japan. Mapping the common S···O and S···N interactions observed by the database analysis, we found that most of the S···X interactions make clusters on the dendrogram. The results suggested a possible role of S···X interactions in molecular evolution of proteins.

### 5.2. Ribonuclease A (RNase A)

**Figure 9:**
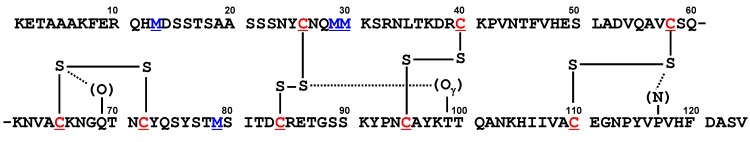
The amino acid sequence of bovine RNase A with notations of common S···X interactions.

RNase A [66] is a typical globular protein of 124 amino acid residues having four SS bonds (C40–C95, C65–C72, C26–C84, and C58–C110) in the native state. Its biological functions as well as the three-dimensional structure and the folding pathways have been extensively studied [67,68]. According to the standardized statistical analysis method [20], close S···X contacts in the structures belonging to the RNase A domain were thoroughly sought. The 43 high-resolution (≤2.0 Å) structures were extracted from the protein data bank, and two common S···O interactions,* i.e.*, S(C26)···O_γ_(T99) and S(C65)···O(Q69), and one common S···N interaction, *i.e.*, S(C58)···N(P117), were characterized. The locations along the amino acid sequence are shown in Figure 9. The SS loop of C65–C72 is one of the important sites that fold in the beginning of the oxidative folding [69] and gives significant thermodynamic stability to the native structure [70]. The presence of the S(C65)···O(Q69) interaction in this loop may be responsible in part for the stability.

In some RNase A structures complexed with a substrate, close S···X contacts between the S atom of the C65 residue and the substrate were found. Examples are S(C65)···O(ADT) in 8RSA [71], S(C65)···N(PAP) in 1AFK [72], S(C65)···N(ATR) in 1AFL [72], and S(C65)···N(PUA) in 1QHC [73]. These interactions would be cooperatively stabilized by the S(C65)···O(Q69) interaction.

### 5.3. Insulin

**Figure 10:**
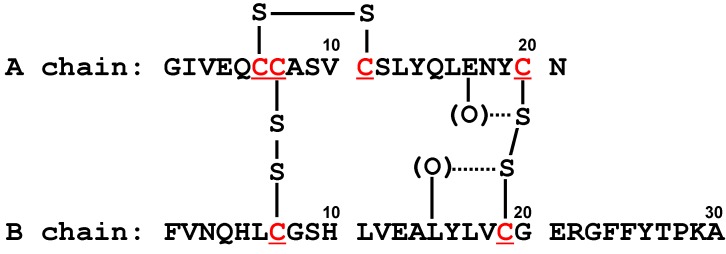
The amino acid sequence of bovine insulin with notations of common S···X interactions.

Insulin [74] is a peptide hormone composed of two peptides (A and B chains), which are connected together by two SS bridges. Another SS bond is present in the A chain. For this model, two common S···O interactions, *i.e.*, S(C20)···O(E17) and S(C19)···O(L15), were found within A and B chains, respectively, among the 23 high-resolution (≤2.0 Å) structures. Figure 10 shows the locations of the interactions. By the complex formation with a ligand, the common S···O interactions were not disrupted, while new S···O interactions were emerged in the A chain; S(C6)···O(I2), S(C7)···O(V3), and S(C11)···O(Q5). These allosteric effects may possibly contribute to the function of insulin. 

### 5.4. Lysozyme

**Figure 11:**
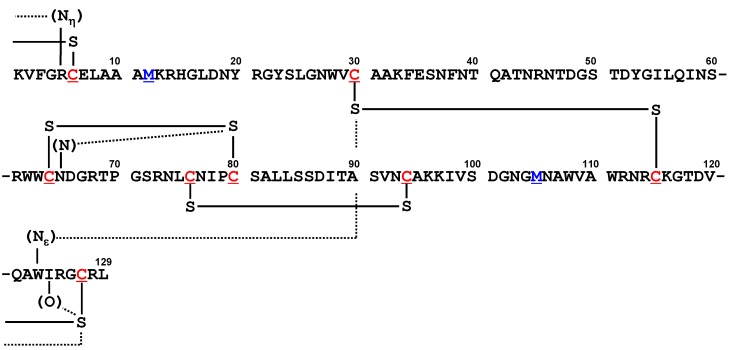
The amino acid sequence of hen egg white lysozyme with notations of common S···X interactions.

Lysozyme [75] consists of 129 or 130 amino acid residues with four SS bonds in the native state. A large number of the structures with various ligand have been registered in the Protein Data Bank. We selected 28 high-resolution (≤1.5 Å) structures from the lysozyme domain and analyzed close S···X (X=O, N, and S) contacts involved in the structures. One common S···O interaction, *i.e.*, S(C127)···O(I124), and three common S···N interactions, *i.e.*, S(C30)···N_ε_(W123), S(C80)···N(N65), and S(C127)···N_η_(R5), were characterized as shown in Figure 11. Although functional or evolutional aspects of these nonbonded interactions are not clear, it is notable that they are not disrupted by the ligand binding like the S···O and S···N interactions found in the other model proteins. 

## 6. Method of Database Analysis

Details of the database analysis of the S···X interactions in PLA_2_ and sPLA_2_ were described in reference [20]. Similar methods were employed for the analysis of the S···X interactions in RNase A, insulin, and lysozyme. For RNase A, 43 high-resolution structures were selected; 1A5P, 1AFK, 1AFL, 1AFU, 1AQP, 1BEL, 1C8W, 1C9V, 1C9X, 1DY5, 1EIC, 1EID, 1EIE, 1F0V, 1FS3, 1JN4, 1JVU, 1LSQ, 1QHC, 1RBW, 1RBX, 1RCA, 1RND, 1RNM, 1RNN, 1RNO, 1RNQ, 1RNW, 1RNX, 1RNY, 1RNZ, 1RPG, 1RUV, 1XPS, 1XPT, 3RN3, 3RSD, 3RSK, 3RSP, 4RSD, 8RAT, 8RSA, and 9RSA. Similarly, 23 high-resolution structures were selected for insulin; 1B17, 1B18, 1B19, 1B2A, 1B2B, 1B2C, 1B2D, 1B2G, 1BEN, 1EV3, 1G7A, 1G7B, 1GUJ, 1M5A, 1MSO, 1TRZ, 1ZEG, 1ZEH, 1ZNI, 2TCI, 3INS, 4INS, and 9INS, and 28 high-resolution structures were selected for lysozyme; 135L, 193L, 194L, 1A2Y, 1AKI, 1HF4, 1IEE, 1IWT, 1IWU, 1IWV, 1IWW, 1IWX, 1IWY, 1IWZ, 1JSE, 1JSF, 1JWR, 1LJN, 1LKS, 1LZ1, 1LZ3, 1LZB, 1LZR, 1QIO, 1REX 2IHL, 3LZT, and 4LZT.

## 7. Conclusions

The three-dimensional structure, hence the function, of a protein is controlled by the interplay of a number of weak nonbonded interactions, such as hydrogen bond, van der Waals forces, and hydrophobic interaction. According to the results from the database analyses and theoretical calculation summarized in this review, it would be concluded that hypervalent S···X interactions are also a member of such weak interactions. 

Sulfur-containing functional groups of cystine (an SSC group) and methionine (a CSC group) were previously considered to be just hydrophobic moieties in protein structures, but they are indeed able to form specific nonbonded interactions with nearby polar non-hydrogen atoms (X = O, N, S) in folded proteins. A unique directionality of the S···X interactions (see Figure 2) would be largely controlled by the orbital interaction between the interacting atoms, while a significant contribution from the electron correlation seems to be important for the stability according to the *ab initio* calculation. For four particular proteins, *i.e.*, PLA_2_, RNase A, insulin, and lysozyme, unique S···X interactions have been characterized, and some were suggested to play roles in the stability of the native structures and the functions to some extent.

Finally, the statistical analyses using the both protein and organic molecule structure databases demonstrated that the order of the strength of the factors that control molecular structures in the solid state can be expressed as shown in Figure 7. The order will be useful for versatile fields of chemistry, such as development of advanced materials built by molecular assembly, molecular design of sulfur-containing ligands or medicines, and protein engineering. The statistical method employed for the S···X interactions, *i.e.*, integration of the database analyses and theoretical calculation, will be useful for characterization of other weak nonbonded interactions hidden in molecules as well as protein structures. 

## Supplementary Materials

Supplementary materials can be accessed at: http://www.mdpi.com/1420-3049/17/6/7266/s1.
