# Supplementary material for: Hypervalent Nonbonded Interactions of a Divalent Sulfur Atom. Implications in Protein Architecture and the Functions
Source: Molecules. 2012 Jun 13;17(6):7266–83. doi: 10.3390/molecules17067266 (PMC6269016; doi:10.3390/molecules17067266)
Supplement: Supplementary file 1 [file molecules-17-07266-s001.pdf]

Molecules **2012**, *17*, 7266-7283; doi:10.3390/molecules17067266

OPEN ACCESS

molecules

ISSN 1420-3049

www.mdpi.com/journal/molecules

Review

Hypervalent Nonbonded Interactions of a Divalent Sulfur Atom. Implications in Protein Architecture and the Functions

Michio Iwaoka * and Noriyoshi Isozumi

Department of Chemistry, School of Science, Tokai University, Kitakaname, Hiratsuka-shi, Kanagawa 259-1292, Japan

* Author to whom correspondence should be addressed; E-Mail: miwaoka@tokai.ac.jp; Tel.: +81-463-58-1211; Fax: +81-463-50-2094.

Received: 21 May 2012; in revised form: 6 June 2012 / Accepted: 8 June 2012 / Published: 13 June 2012

Supporting Information

Table S1. Nonbonded S•••X interactions found in RNase A.
Table S2. Nonbonded S•••X interactions found in insulin.
Table S3. Nonbonded S•••X interactions found in lysozyme.

Table S1. Nonbonded S•••X interactions found in RNase A.

Distances (r) and the relative distances [d = r − vdw(S) − vdw(X)] are given in Å.

			S(C26)•••Oγ(T99)		S(C65)•••O(Q69)		S(C58)•••N(P117)	
PDBID	Resolution	Chain	r(S•••O)	d(S•••O)	r(S•••O)	d(S•••O)	r(S•••N)	d(S•••N)
1a5p	1.6				3.458	0.138	3.484	0.134
1afk	1.7	A	3.109	−0.211			3.485	0.135
1afk	1.7	B	3.128	−0.192				
1afl	1.7	A	3.177	−0.143	3.456	0.136	3.512	0.162
1afl	1.7	B	3.230	−0.090	3.501	0.181		
1afu	2	A	3.253	−0.067				
1afu	2	B	3.142	−0.178				
1aqp	2	_					3.443	0.093
1bel	1.6	A	3.259	−0.061			3.444	0.094
1c8w	1.8	A	3.240	−0.080	3.426	0.106	3.499	0.149
1c9v	1.7	A	3.176	−0.144	3.404	0.084	3.498	0.148
1c9x	1.8	A	3.179	−0.141	3.407	0.087		
1dy5	0.87	A	3.115	−0.206	3.471	0.151	3.493	0.143
1dy5	0.87	B	3.050	−0.270				
1eic	1.4	A	3.222	−0.098	3.515	0.195	3.393	0.043
1eid	1.4	A	3.283	−0.037	3.210	−0.110	3.464	0.114
1eie	1.4	A	3.202	−0.118	3.517	0.197	3.414	0.064
1f0v	1.7	A	3.150	−0.170				
1fs3	1.4	A	3.272	−0.048			3.407	0.057
1jn4	1.8	A	3.146	−0.174	3.446	0.126	3.461	0.111
1jn4	1.8	B	3.086	−0.234				
1jvu	1.78	A	3.221	−0.100	3.513	0.193	3.509	0.159
1jvu	1.78	B	3.255	−0.065				
1lsq	1.9	A	3.155	−0.165	3.316	−0.004		
1lsq	1.9	B	3.206	−0.114	3.346	0.026		
1qhc	1.7	A	3.062	−0.258	3.507	0.187	3.455	0.105
1qhc	1.7	B	3.153	−0.167				
1rbw	1.69	_	3.272	−0.048	3.384	0.064	3.484	0.134
1rbx	1.69	_	3.332	0.012	3.493	0.173	3.514	0.164
1rca	1.9	_	3.381	0.061	3.355	0.035	3.532	0.182
1rnd	1.5	_	3.276	−0.044	3.427	0.107	3.440	0.090
1rnm	2	E	3.109	−0.211			3.482	0.132
1rnn	1.8	E	3.168	−0.153			3.534	0.184
1rno	1.9	_	3.262	−0.058			3.533	0.183
1rnq	2	_	3.306	−0.014				
1rnw	1.8	_	3.248	−0.072			3.529	0.179
1rnx	1.9	_	3.268	−0.052				
1rny	2	_	3.292	−0.029	3.415	0.095		
1rnz	1.9	_	3.239	−0.081				
1rpg	1.4	_	3.264	−0.056	3.459	0.139	3.519	0.169
1ruv	1.3	_	3.177	−0.143	3.426	0.106	3.468	0.118
1xps	1.8	A	3.259	−0.061			3.419	0.069
1xps	1.8	B	3.279	−0.041	3.213	−0.107		
1xpt	1.9	A	3.343	0.023				
1xpt	1.9	B	3.203	−0.117				
3rn3	1.45	_	3.217	−0.103	3.405	0.085	3.469	0.119
3rsd	1.6	_	3.177	−0.143	3.339	0.019	3.477	0.127
3rsk	2	_	3.096	−0.224			3.508	0.158
3rsp	1.7	_	3.207	−0.114	3.475	0.155		
4rsd	1.6	_	3.219	−0.101	3.145	−0.175	3.504	0.154
8rat	1.5	_	3.160	−0.160	3.367	0.047	3.415	0.065
8rsa	1.8	A	3.127	−0.193			3.479	0.129
8rsa	1.8	B	3.128	−0.192	3.399	0.079	3.524	0.174
9rsa	1.8	A	3.066	−0.254			3.323	−0.027
9rsa	1.8	B	3.184	−0.136	3.353	0.033	3.490	0.140

Table S2. Nonbonded S•••X interactions found in insulin.

Distances (r) and the relative distances [d = r − vdw(S) − vdw(X)] are given in Å.

			A chain S(C20)•••O(E17)		S(C6)•••O(I2)		S(C7)•••O(V3)		S(C11)•••O(Q5)		B chain S(C19)•••O(L15)	
PDBID	Resolution	Chain	r(S•••O)	d(S•••O)	r(S•••O)	d(S•••O)	r(S•••O)	d(S•••O)	r(S•••O)	d(S•••O)	r(S•••O)	d(S•••O)
1BEN	1.4	C					3.342	0.022				
1EV3	1.78	A							3.496	0.176		
1EV3	1.78	C	3.475	0.155								
1G7A	1.2	A	3.503	0.183					3.319	−0.001		
1G7A	1.2	C	3.513	0.193					3.400	0.080		
1G7A	1.2	G	3.420	0.100					3.409	0.089		
1G7B	1.3	A	3.493	0.173					3.310	−0.010		
1G7B	1.3	C	3.472	0.152					3.513	0.193		
1G7B	1.3	E							3.238	−0.082		
1G7B	1.3	G	3.405	0.085					3.135	−0.185		
1GUJ	1.62	A	3.336	0.016								
1GUJ	1.62	C	3.335	0.015								
1M5A	1.2	A			3.346	0.026						
1M5A	1.2	C	3.478	0.158								
1MSO	1	A	3.344	0.024	3.321	0.001						
1TRZ	1.6	A	3.508	0.188					3.479	0.159		
1TRZ	1.6	C					3.357	0.037				
1ZEG	1.6	A	3.449	0.129								
1ZEH	1.5	A	3.418	0.098								
1ZNI	1.5	A	3.503	0.183			3.183	−0.138	3.478	0.158		
1ZNI	1.5	C							3.454	0.134		
2TCI	1.8	A			3.515	0.195	3.119	−0.201				
3INS	1.5	A			3.316	−0.004						
4INS	1.5	A			3.267	−0.053						
1B17	1.7	B									3.388	0.068
1B18	1.8	B									3.359	0.039
1B19	1.8	B									3.372	0.052
1B2A	1.7	B									3.373	0.053
1B2B	1.8	B									3.356	0.036
1B2C	1.8	B									3.400	0.080
1B2D	1.7	B									3.378	0.058
1B2G	1.8	B									3.409	0.089
1BEN	1.4	B									3.359	0.039
1BEN	1.4	D									3.362	0.042
1EV3	1.78	B									3.380	0.060
1EV3	1.78	D									3.294	−0.026
1G7A	1.2	B									3.331	0.011
1G7A	1.2	D									3.254	−0.066
1G7A	1.2	F									3.302	−0.018
1G7A	1.2	H									3.289	−0.031
1G7B	1.3	B									3.302	−0.018
1G7B	1.3	D									3.267	−0.053
1G7B	1.3	F									3.302	−0.019
1G7B	1.3	H									3.281	−0.039
1GUJ	1.62	B									3.208	−0.112
1GUJ	1.62	D									3.180	−0.140
1M5A	1.2	B									3.325	0.005
1M5A	1.2	D									3.351	0.031
1MSO	1	B									3.400	0.080
1MSO	1	D									3.403	0.083
1TRZ	1.6	B									3.305	−0.015
1TRZ	1.6	D									3.345	0.025
1ZEG	1.6	B									3.178	−0.142
1ZEG	1.6	D									3.256	−0.064
1ZEH	1.5	B									3.178	−0.142
1ZEH	1.5	D									3.253	−0.067
1ZNI	1.5	B									3.333	0.013
1ZNI	1.5	D									3.334	0.014
2TCI	1.8	B									3.323	0.003
2TCI	1.8	D									3.283	−0.037
3INS	1.5	B									3.395	0.075
3INS	1.5	D									3.454	0.134
4INS	1.5	B									3.443	0.123
4INS	1.5	D									3.478	0.158
9INS	1.7	B									3.429	0.109

Table S3. Nonbonded S•••X interactions found in lysozyme.

Distances (r) and the relative distances [d = r − vdw(S) − vdw(X)] are given in Å.

			S(C127)•••O(I124)		S(C30)•••Nε(W123)		S(C80)•••N(N65) * **		S(C127)•••Nη(R5)	
PDBID	Resolution	Chain	r(S•••O)	d(S•••O)	r(S•••N)	d(S•••N)	r(S•••N)	d(S•••N)	r(S•••N)	d(S•••N)
135L	1.3	_	3.464	0.144	3.307	−0.043	3.493	0.143	3.492	0.142
193L	1.33	_	3.260	−0.060	3.320	−0.030	3.547	0.197	3.323	−0.027
194L	1.4	_	3.250	−0.070	3.287	−0.063	3.505	0.155	3.290	−0.060
1A2Y	1.5	C					3.415	0.065		
1AKI	1.5	_	3.451	0.131	3.371	0.021				
1HF4	1.45	A	3.390	0.070	3.227	−0.123				
1HF4	1.45	B			3.295	−0.055	3.526	0.176		
1IEE	0.94	A	3.426	0.106	3.334	−0.016	3.519	0.169		
1IWT	1.4	A							3.264	−0.086
1IWU	1.4	A							3.256	−0.094
1IWV	1.4	A							3.309	−0.041
1IWW	1.4	A					3.548	0.198	3.322	−0.028
1IWX	1.4	A							3.211	−0.139
1IWY	1.4	A					3.521	0.171	3.321	−0.029
1IWZ	1.4	A	3.510	0.190			3.542	0.192	3.530	0.180
1JSE	1.12	_	3.459	0.139	3.338	−0.012	3.522	0.172		
1JSF	1.15	_	3.501	0.181			3.457	0.107	3.377	0.027
1JWR	1.4	A	3.506	0.186					3.327	−0.023
1LJN	1.19	A	3.491	0.171	3.338	−0.012	3.535	0.185		
1LKS	1.1	_	3.382	0.062	3.510	0.160			3.536	0.186
1LZ1	1.5	_					3.511	0.161	3.437	0.087
1LZ3	1.5	_			3.334	−0.016	3.535	0.185	3.525	0.175
1LZB	1.5	_	3.319	−0.002	3.292	−0.058	3.492	0.142	3.472	0.122
1LZR	1.5	_	3.482	0.162			3.511	0.161	3.418	0.068
1QIO	1.2	A	3.397	0.077	3.390	0.040				
1REX	1.5	_					3.403	0.053	3.522	0.172
2IHL	1.4	_	3.411	0.091	3.473	0.123	3.471	0.121		
3LZT	0.92	_	3.285	−0.035	3.514	0.164	3.546	0.196	3.474	0.124
4LZT	0.95	_	3.388	0.068	3.486	0.136	3.536	0.186		

* S(C80)•••N(D66) for 135L, 1HF4, 1JSE, 1LJN, 2IHL. ** S(C81)•••N(N66) for 1IWW, 1IWY, 1IWZ, 1JSF, 1LZ1, 1LZR, 1REX.
